# Effectiveness of Clear Aligners on Sequential Maxillary Molar Distalization: Discrepancy between Treatment Goal and Outcome

**DOI:** 10.3390/jcm13144216

**Published:** 2024-07-19

**Authors:** Jatuphol Mamani, Chidchanok Sessirisombat, Hitoshi Hotokezaka, Noriaki Yoshida, Irin Sirisoontorn

**Affiliations:** 1 Department of Clinical Dentistry, Walailak University International College of Dentistry (WUICD), 87 Ra-nong 2 Road, Dusit, Bangkok 10300, Thailand; 2 Department of Orthodontics and Dentofacial Orthopedics, Nagasaki University Graduate School of Biomedical Sciences, 1-7-1 Sakamoto, Nagasaki 852-8588, Japan

**Keywords:** molar distalization, sequential molar distalization, clear aligner, invisalign, orthodontic treatment, tooth movement

## Abstract

**Objectives:** The purpose of this preliminary study was to determine the differences between planned and actual maxillary molar movements after the completion of treatment with an initial set of clear aligners including sequential maxillary molar distalization. **Methods:** The data records of 14 non-growing patients who completed orthodontic treatment with sequential maxillary molar distalization using clear aligners were retrospectively evaluated (*n*= 14, 4 males and 10 females, 33.61 ± 8.57 years). Data on planned tooth movements were obtained from ClinCheck software (ClinCheck Pro version 5.3). The amounts of actual tooth movements were obtained by performing superimposition of lateral cephalograms taken before and after treatment. The amounts of distal translation and tipping between planned and actual maxillary molar movements were compared with the paired Student’s *t*-test. **Results:** The statistically significant differences between planned and actual translation movements of maxillary first and second molars were shown after completing treatment with the first series of aligners (*p* < 0.05). The average actual amount of molar distalization on maxillary first molars was less than the planned amount by 1.32 ± 0.42 mm. Similarly, the average actual amount of molar distalization on maxillary second molars was less than the planned amount by 1.57 ± 0.45 mm. The accuracy for molar distalization, namely, the percentage of actual distal translation to planned movement, was 40.11% for maxillary first molars and 35.39% for maxillary second molars. However, the difference between the planned and actual angulation movements was not significant (*p* > 0.05). **Conslusions:** In conclusion, the amounts of actual distal translation of maxillary molars through the utilization of clear aligners were significantly lower than planned. However, there were no statistically significant differences between the degrees of actual and planned molar angulation movement.

## 1. Introduction

Class II malocclusion is a prevalent orthodontic condition characterized by an abnormal sagittal relationship between the maxillary and mandibular arches, often involving an anteriorly positioned maxilla relative to the mandible [1]. It is one of the most common malocclusions observed in clinical practice, affecting a significant portion of the global population [2]. Epidemiological studies have consistently reported varying prevalence rates of class II malocclusion across different populations. For instance, studies have documented prevalence rates ranging from 15% to 35%, depending on the specific criteria used to define and classify class II malocclusion [3,4].

Class II malocclusions can be treated using a variety of orthodontic techniques. Maxillary molar distalization was one of the methods that was commonly used to improve the molar relationship from class II to class I and obtain sufficient space for incisor retraction during the early stages of treatment [5,6]. Kinzinger et al. reported that using appliances along with molar distalization in the sagittal direction resulted in a small amount of vertical movement. In addition, the movement of the molars during distalization was not limited purely to translation because force was applied coronally to the center of resistance. The undesirable forces and outcomes differed depending on the type of appliance utilized throughout the treatment procedures [7]. Moreover, the distalization movement of molars may be affected by the degree of anchorage loss during the retraction of the premolars, canines, and incisors [5]. Anchorage loss is more prevalent in males, adolescents, class II malocclusion patients, and those who have had their maxillary premolars extracted. Anchorage loss begins with the mesial tipping of the first molar, and alterations in the angulation of the first molar are strongly associated with anchorage loss. However, physiological characteristics of patients have a stronger effect on changes in the angulation of the maxillary first molars during orthodontic therapy than treatment-related factors [8].

In recent decades, clear aligner therapy has emerged as a popular alternative to traditional fixed appliances for orthodontic treatment. Clear aligners offer advantages such as improved aesthetics, enhanced patient comfort, and the convenience of removable aligners [9,10]. Clear aligners can predictably treat a wide range of malocclusions. The practitioners must have extensive experience, the ability to perform digital treatment planning, and appropriate treatment protocol applications to successfully treat complex cases [11]. Furthermore, the achieved tooth movement may differ from the planned tooth movement since the ClinCheck program used for planning treatment positions teeth solely by computer manipulation. It does not consider the physical characteristics and limitations of the aligners, or the unique biomechanical responses of each patient’s teeth throughout the treatment process [12].

Several systematic reviews examined the efficacy of aligners in orthodontic treatment for tooth movement [9,13,14,15]. These reviews demonstrated that clear aligners were effective in distalizing maxillary molars, with high predictability when a movement of at least 1.5 mm was prescribed. Systematic reviews on the accuracy of distal translation movement with clear aligners showed an overall accuracy of approximately 70–88% for distalizing the buccal cusps of the first and second molars. This accuracy was influenced by several factors, including the use of auxiliary devices such as attachments, interarch elastics, and temporary anchorage devices (TADs), which significantly enhanced movement precision and control. However, there were discrepancies between predicted and actual movements, often necessitating adjustments to the treatment plan during the course of treatment due to discrepancies between the planned and actual tooth movements to achieve the desired outcomes and ensure proper alignment and function of the teeth.

The aim of this preliminary study was to determine if there are significant differences between planned and actual maxillary molar movements after the completion of sequential maxillary molar distalization by an initial set of aligners. This study was conducted to enhance the understanding of clear aligner treatment with the use of elastics for the correction of class I and class II malocclusion. Furthermore, by analyzing the treatment outcomes after the completion of the initial set of aligners, the true performance of the aligners was determined.

## 2. Materials and Methods

### 2.1. Sample Size Calculation

G* Power 3.1 software (G*Power, Heinrich-Heine-Universität Düsseldorf, Düsseldorf, Germany) indicated that a minimum sample size of *n* = 15 was required for this preliminary study to guarantee sufficient statistical power (0.8), with α = 0.05 and an effect size of 0.8. According to Cohen’s conventions, an effect size of 0.8 is considered large, indicating a substantial difference between groups. The decision to use this value was based on the need to detect meaningful clinical differences with adequate statistical power. Given the nature of this study and the anticipated differences based on prior research [16], an effect size of 0.8 was deemed appropriate to ensure that this study could reliably identify significant effects, if they exist. This choice balanced the need for a reasonable sample size with the ability to detect clinically important differences. Although the ideal sample size for this study was calculated to be 15 to achieve the desired statistical power, only 14 participants were included in the final analysis due to constraints such as the number of samples meeting all criteria and data completeness. Despite this, this study was conducted with a rigorous methodology to ensure the reliability and validity of the results.

### 2.2. Participants and Study Setting

The data records of 14 patients who received orthodontic treatment with Invisalign were retrospectively assessed (N = 14, 4 males and 10 females, mean age and SD 33.61 ± 8.56, range from 20 to 49). All patients were treated by a skilled orthodontist with considerable experience in treating patients with clear aligners in a private practice in Bangkok, Thailand. The treatment started in 2018 or later and met the inclusion/exclusion criteria listed below:

Inclusion criteria

(1) Age between 19 and 60 years old
(2) Skeletal type I or II with class II molar relationship
(3) Non-extraction treatment plan (except for third molars)
(4) Treatment aimed to achieve distalization of the maxillary molar by at least 1.5 mm
(5) Patients had been prescribed sequential molar distalization and class II elastic in their treatments
(6) Completion of the first series of aligners
(7) Absence of maxillary third molars during treatment
(8) Cephalograms properly positioned, clear, and symmetrical, with visible key landmarks and soft tissue profiles

Exclusion criteria

(1) Patients prescribed skeletal anchorage in their treatment

(2) Patient charts indicating poor compliance, including not wearing aligners as prescribed, missing follow-up appointments, and skipping the use of elastics

(3) Unilateral distalization

(4) Untreated periodontal disease

(5) Dental restorations on maxillary molars during treatment with the first series of aligners

### 2.3. Treatment Protocol

All ClinCheck plans were prescribed in order to obtain sequential molar distalization, with class II elastics attached to buttons bonded to the buccal surfaces of upper canines and lower first molars. Class II elastics were consistently used full-time with 1/4-inch, 4.5-ounce elastics (American Orthodontics Corporate, Sheboygan, WI, USA). The ClinCheck plans were created according to the orthodontist’s preferences, without restrictions on attachment placement. A 7-day aligner change protocol was used throughout the duration of treatment.

### 2.4. Pretreatment and Posttreatment Lateral Cephalograms

**Figure 1:**
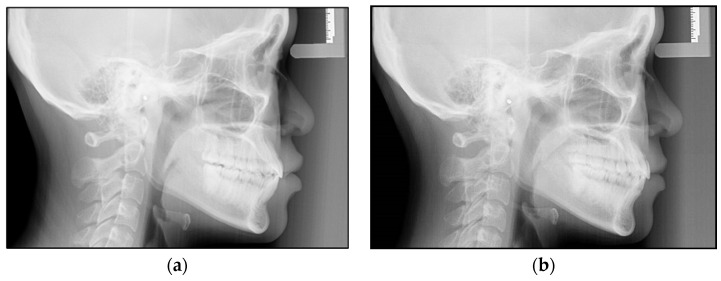
(**a**,**b**) Lateral cephalometric radiographs of a patient at the beginning of the treatment (**a**) and after treatment with the first series of Invisalign (**b**).

In total, 28 lateral cephalometric radiographs of the subjects were considered in this study. All the cephalometric radiographs were taken using a digital cephalometer (Orthopanmograph OP 200D, Instrumentarium Dental, Tuusula, Finland). The digital images were stored in a computer database with the manufacturer’s software and imported into Adobe Photoshop CS software (version 20). The software was used to resize the digital photos to a 1:1 ratio, and the images were printed using a 4800 dpi inkjet color printer (Canon PIXMA G4010, Canon Inc., Tokyo, Japan) on 180 gsm glossy inkjet photo paper made for high-quality photographic images. The cephalometric radiographs that were collected at the beginning were defined as Pretreatment (T0) and those at the end of the first series of Invisalign treatment were defined as Posttreatment (T1) (Figure 1a,b).

### 2.5. ClinCheck Software

**Figure 2:**
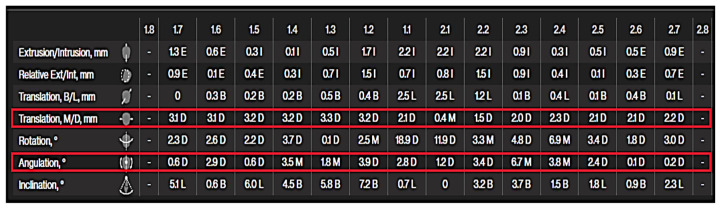
Tooth movement table displaying the mesiodistal translation and mesiodistal angulation that were used in this study.

ClinCheck software (ClinCheck Pro version 5.3) is the Invisalign treatment software developed to provide precise control over tooth position and assist dental practitioners in achieving treatment goals. This software generates an accurate three-dimensional model of a patient’s teeth, allowing practitioners to effectively plan and customize tooth movements. The software simulates every stage of treatment, depicted as a series of aligners or adjustments, and allows practitioners to make real-time modifications to achieve the most favorable outcomes. With the Tooth Movements Table feature, dental practitioners can view all the movements programmed for each tooth based on the crown center or the virtual root apex. The Tooth Movements Table revealed the amount of movement of the first and second maxillary molars in mesiodistal translation and mesiodistal tipping (Figure 2).

### 2.6. Ethical Approval

This retrospective study was ethically approved by the Institutional Review Board, Walailak University, Thailand (approval number: WUEC-20-178-01) on 3 July 2020. The Human Research Ethics Committee of Walailak University granted an exempt status for this project.

### 2.7. Tracing of Cephalograms

The printed cephalogram images were manually traced with a 0.3 mm lead pencil on fine-grain 0.003-inch transparent acetate sheets. All radiographs were traced by a single researcher to minimize discrepancies in the tracing procedures. To decrease the risk of possible error during the tracing of the cephalometric radiographs, the researcher was trained in cephalometric tracing and analysis by an experienced orthodontist. The anterior nasal spine to posterior nasal spine line (ANS-PNS line), which had no significant change with age [17], represented the *X*-axis and the perpendicular line to the ANS-PNS line passing through the posterior side of the pterygomandibular fissure (Ptm) represented the *Y*-axis.

### 2.8. Cephalometric Measurements

**Figure 3:**
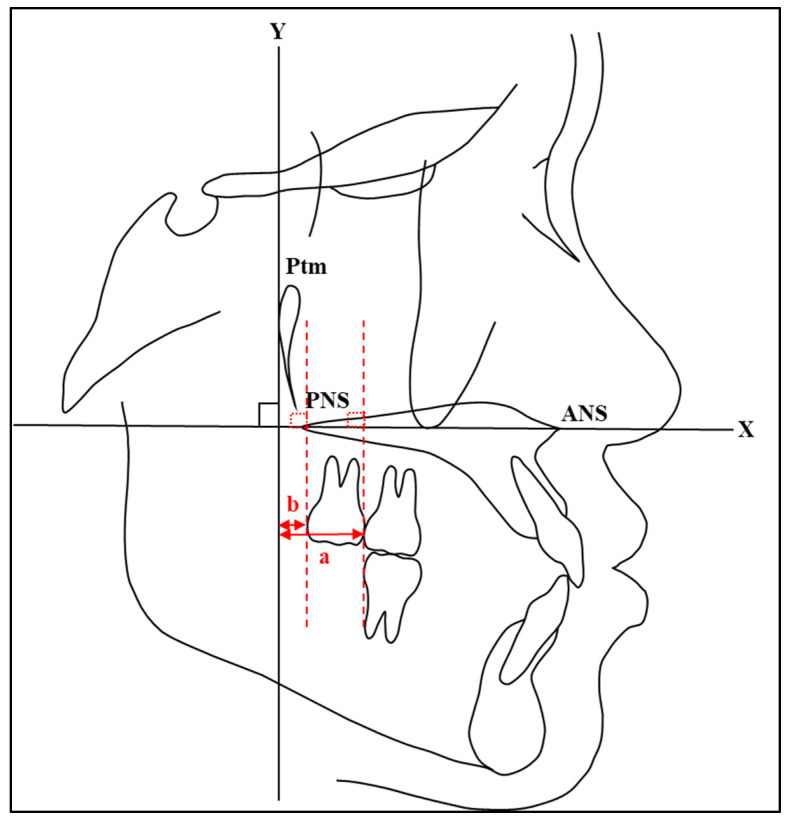
A diagrammatic representation of the cephalometric measurements of maxillary molar positions acquired by measuring the distances between the *Y*-axis and distal convex spots on the crown of first (a) and second (b) maxillary molars.

**Figure 4:**
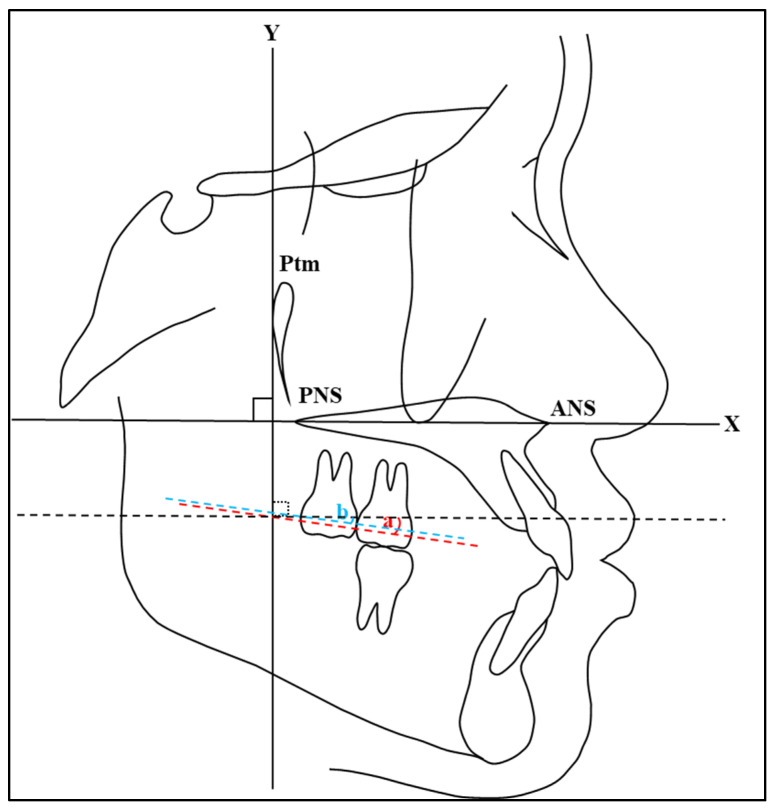
A diagrammatic representation of the cephalometric measurements of maxillary molar angulation acquired by measuring the angle between the *X*-axis and the line passing through between the most mesial and distal convex points on the crown of first (a) and second (b) maxillary molars.

The most convex points on the distal surface of the first and second maxillary molars were used to determine the distal points of the maxillary molars. If there were double contours between the left and right molars, the mean bisector of the distal point was considered for measuring. The measurements of maxillary molar positions were obtained by measuring the distance between the *Y*-axis and distal convex spots on the crown of the first and second maxillary molars using a digital vernier caliper (Mitutoyo-Japan Corp.) (Figure 3). In addition, the measurements of maxillary molar angulations were obtained by measuring the angle between the *X*-axis and the line passing through between the most mesial and distal convex points on the crown of the first and second maxillary molars using a cephalometric protractor (Ormco Corp.) (Figure 4). These measurement methods are consistent with those used in previous studies. For instance, Proffit et al. [1] described similar methods for assessing molar positions and angulations in their comprehensive overview of orthodontic diagnosis and treatment planning. In addition, studies by Roth [18] and McNamara [19] utilized similar approaches for assessing molar positions and angulations using the most convex points as reference landmarks.

**Figure 5:**
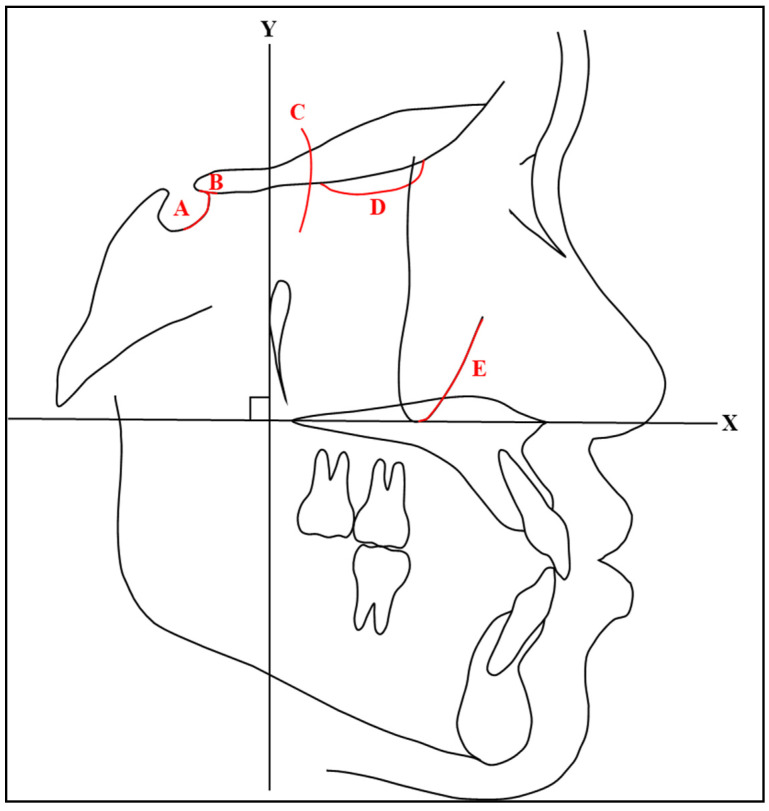
A diagrammatic representation of the superimposed area: (A) anterior wall of sella turcica, (B) Walker’s point, (C) middle cranial fossae, (D) cribriform plate, and (E) anterior contour of the zygomatic process.

Using the structural method, pretreatment and posttreatment radiographs were superimposed on stable anatomical structures. This study used the following landmarks for superimposition: (1) the inner contour of the anterior wall of the sella turcica, (2) the intersection point of the lower contours of the anterior clinoid processes and the contour of the anterior wall of the sella turcica (Walker’s point), (3) the anterior contours of the middle cranial fossae, (4) the contour of the cribriform plate, and (5) the anterior contour of the zygomatic process (Figure 5). These landmarks were based on Bjork’s longitudinal growth experiments with tantalum implants [20] and the study on the growth of the maxilla by Bjork and Skieller [21].

**Figure 6:**
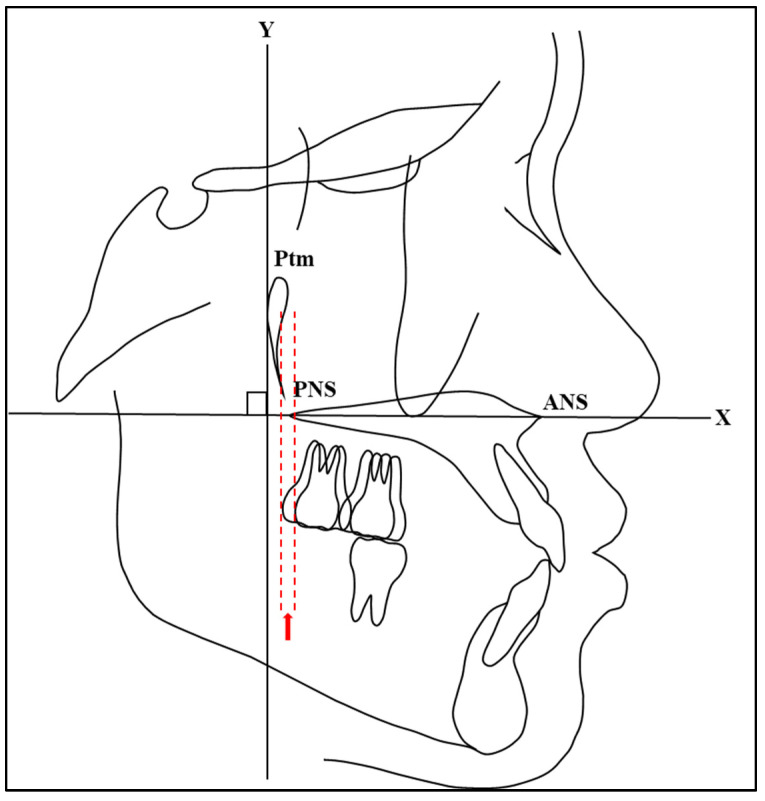
A diagrammatic representation of the distance between distal points of second maxillary molars at T0 and T1.

**Figure 7:**
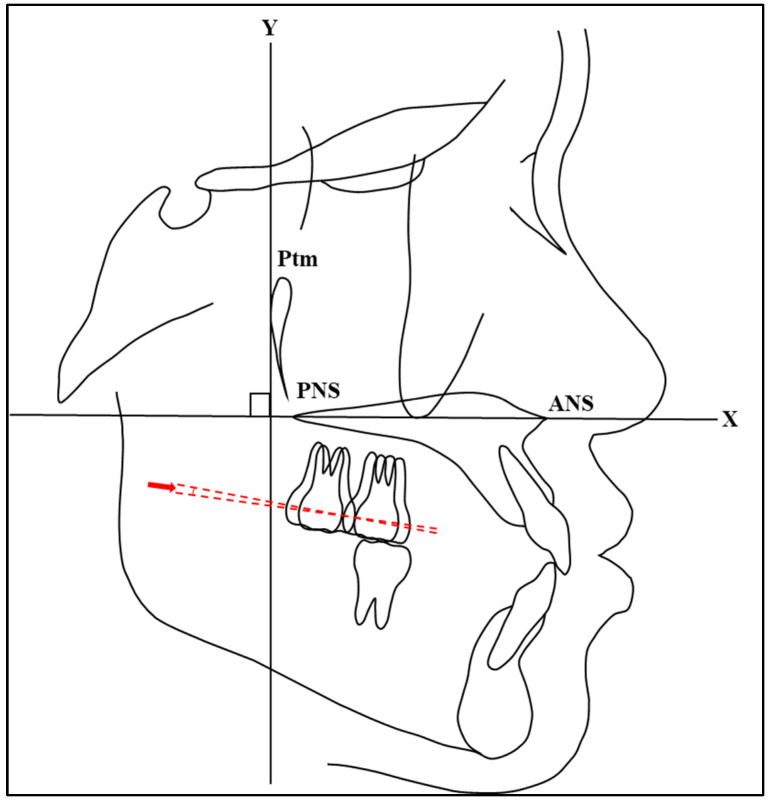
A diagrammatic representation of the angulation between the line passing through between the most mesial and distal convex points on the crown second maxillary molars at T0 and T1.

To evaluate molar distalization, the distances between the distal points from T0 and T1 for the first and second maxillary molars were measured (Figure 6). Distal movement was assigned a positive value, while mesial movement was assigned a negative value. Similarly, the angulations of the molars at T0 and T1 were compared to determine the molars’ tipping (Figure 7). A positive value was applied to distal tipping, whereas mesial tipping was assigned a negative value.

### 2.9. Data Collection from ClinCheck

The ClinCheck program’s tooth movement table for each subject was explored for information regarding the mesiodistal translation and mesiodistal tipping of maxillary molars. Positive values were assigned to distal translation and distal angulation, while negative values were ascribed to mesial translation and mesial angulation. Prior to their use in this study, the data for left and right movements were averaged if they were not equal.

### 2.10. Comparison between Planned and Actual Movements

Planned maxillary molar movements from ClinCheck were compared to actual maxillary molar movements from cephalometric analysis to identify their differences. The difference between planned and actual molar movement was determined by subtracting the distal translation distance of the actual molar movement from that of the planned molar movement. Similarly, the difference in molar angulation was determined by subtracting the distal angulation of the actual molar movement from that of the planned molar movement. Positive values were assigned for distal translation/angulation. Negative values were assigned for mesial translation/angulation.

### 2.11. Statistical

Data analysis was performed using the Statistical Package of Social Sciences (SPSS) version 25 (IBM Corp., Armonk, NY, USA). The Shapiro–Wilk test was used to assess normal distribution [22]. Descriptive statistics were calculated for all variables and presented as means ± standard deviations (SDs), medians, and ranges [23]. A paired Student’s *t*-test was used to identify the differences between planned and actual groups if data had a normal distribution. Conversely, if the data distribution was not normal, the non-parametric Wilcoxon signed-rank test was utilized for statistical analysis. An alpha error of 0.05 was used as the level of statistical significance for all analyses. To investigate intraexaminer reliability, twenty percent of the samples were randomly selected, and the same operator repeated the analysis of both linear and angular measurements one month after the first analysis. Cronbach’s alpha was used to measure intraexaminer reliability [24]. The Cronbach’s alpha coefficient ranged from 0 to 1, where alpha values were described as excellent (0.91–1.00), good (0.81–0.90), good and acceptable (0.71–0.80), acceptable (0.61–0.70), and non-acceptable (0.01–0.60).

## 3. Results

Sample demographic information is shown in Table 1. The sample group comprised 14 adult patients (4 male, 10 female), with ages ranging from 20 to 49 years, with a mean age ± SD of 33.61 ± 8.56 years. The average number of aligners was 49.14 ± 14.02. Intraexaminer reliability showed excellent reliability ranging from 0.910 to 0.911 for linear measurements and 0.898 to 0.996 for angular measurements.

**Figure 8:**
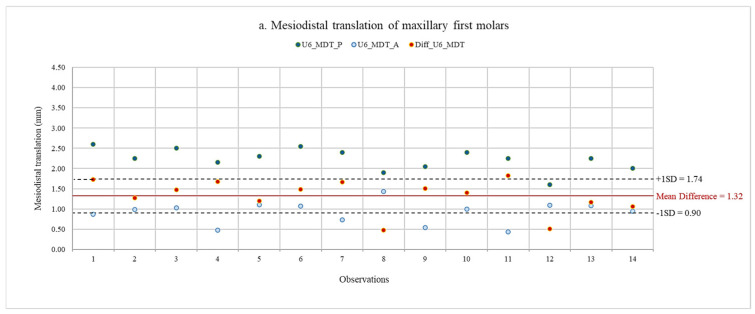

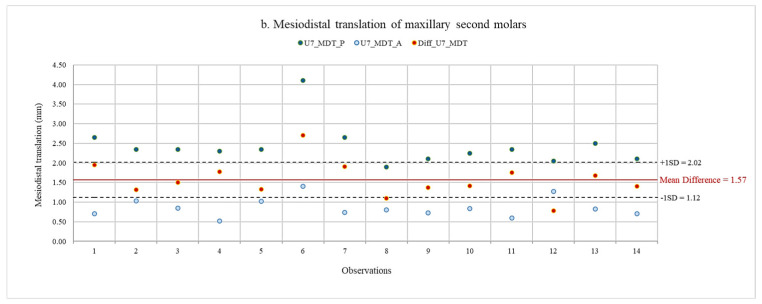
Data of planned and actual mesiodistal translation, with mean difference and standard deviation of maxillary first and second molars. (**a**) Mesiodistal translation of maxillary first molars. (**b**) Mesiodistal translation of maxillary second molars. U6, maxillary first molar; U7, maxillary second molar; MDT_P, planned mesiodistal translation; MDT_A, actual mesiodistal translation; Diff, difference between planned and actual movements.

**Figure 9:**
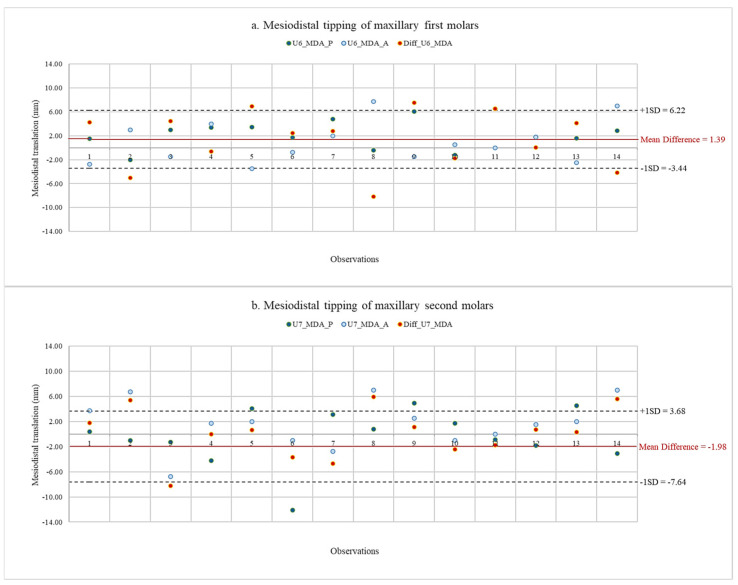
Data of planned and actual mesiodistal tipping with mean difference and standard deviation of maxillary first and second molars. (**a**) Mesiodistal tipping of maxillary first molars. (**b**) Mesiodistal tipping of maxillary second molars. U6, maxillary first molar; U7, maxillary second molar; MDA_P, planned mesiodistal tipping; MDA_A, actual mesiodistal tipping; Diff, difference between planned and actual movements.

Using scatter diagrams, the data of planned and actual movements of maxillary first and second molars, as well as their discrepancy, are shown in Figure 8 and Figure 9. Descriptive statistics of the planned and actual maxillary molar movements are presented in Table 2. Since the data distribution was normal for all tooth movements, comparisons between planned and actual maxillary molar movements were made using a paired Student’s *t*-test.

The results for translation and angulation movements are presented in Table 3. A negative value indicated that the actual values showed greater distal movements than the planned values. The statistically significant differences between planned and actual translation movements of maxillary first and second molars were shown after completing treatment with the first series of aligners. The average actual amount of molar distalization on maxillary first molars was less than the planned amount by 1.32 ± 0.42 mm. Similarly, the average actual amount of molar distalization on maxillary second molars was less than the planned amount by 1.57 ± 0.45 mm. The ratios of actual distal translation to planned movement were 40.11 for maxillary first molars and 35.39 for maxillary second molars. However, the difference between the planned and actual angulation movements was not significant. Maxillary first molars had lower amounts of distal tipping in mean actual mesiodistal angulation, with a difference of 1.39° ± 4.83°. On the other hand, a greater amount of distal tipping was found in actual mesiodistal angulation, with a difference of 1.98° ± 5.66°.

## 4. Discussion

Since the Invisalign appliance was introduced in the late 1990s, this treatment system has achieved significant advancements in terms of treatment planning methods, materials, and manufacturing. The system’s capabilities are continuously making progress, and improvements are being introduced nearly every year [25]. Therefore, the subjects used in this study were selected from patients who underwent orthodontic treatment with clear aligners from 2018 onwards to make the effectiveness of the aligners as similar as possible to the present system. Some studies investigated the dento-skeletal effects of maxillary molar distalization therapy in growing patients [26,27]. They reported that the molars ended up being located in an even more mesial position than before treatment, including with molar distalization. Therefore, growing subjects were excluded from this study in order to minimize any discrepancies that could have occurred in the experimental results. Class II intermaxillary elastics were used to decrease anchorage loss during distalization of the maxillary molars [28]. Significant dentoalveolar changes were primarily responsible for the overjet correction following the use of intermaxillary elastics, including the retroclination of maxillary incisors, proclination of lower incisors, and clockwise rotation of the occlusal plane. As a result, the upper lip slightly retruded and the lower lip protruded [29]. In addition, when the attachments were placed on the distalized teeth of non-growing patients requiring 2–3 mm of bodily maxillary molar distalization, they seemed to be effective in minimizing distal crown tipping and preventing molar extrusion, anterior anchorage loss, and undesirable changes in lower facial height [30].

In this study, the actual maxillary molar translation movements differed significantly from those planned. The average actual distal movement of the maxillary first molar was 0.91 mm and the average actual distal movement of the maxillary second molar was 0.86 mm, approximately 40.11% and 35.39% of the planned distal movements, respectively. The results from the present study are not in agreement with the results obtained by Simon et al. [31], D’Antò et al. [32], and Ravera et al. [16]. Simon et al. reported that the distalization of maxillary molars was the most effective movement when distalization greater than 1.5 mm was prescribed, achieving a high accuracy of 88.4% in molar distalization when using attachments. Similarly, D’Antò et al. reported that the accuracy of buccal cusp distalization was 69.3% for the first molar and 75.2% for the second molar. However, this evaluation was performed immediately after the completion of the sequential distalization of first and second molars. Also, the following anchorage loss in the posterior region that occurred due to the reciprocal force reacting to anterior teeth retraction was not taken into consideration. A previous study by Ravera et al. found that Invisalign aligners were effective in distalizing maxillary molars in non-growing patients when evaluated at the end of the treatment. The amount of distal movement was 2.25 mm for the first molar and 2.52 mm for the second molar. However, the outcomes should be measured at the end of the first series of aligners, not the end of treatment. With this method, it is possible to acquire a realistic assessment of tooth movements during a fixed period of time.

The distalization of maxillary molars using clear aligners revealed no statistically significant difference between planned and actual molar tippings in our investigation. However, the mean actual distal tipping of the maxillary first molar was 1.39 degrees less than planned, while the mean distal tipping of the maxillary second molar was 1.98 degrees greater than planned. Previous studies by Ravera et al. [16] and Caruso et al. [33] reported an absence of distal tipping when performing upper molar distalization; however, these studies were conducted at the end of treatment. Greater distal tipping on actual second molar movement occurred in this study because the force was delivered coronally to the center of resistance, leading the crown to move more posteriorly than the root [5]. This phenomenon should have occurred with the maxillary first molar as well; however, the mesial force was applied on the first molar crown during the retraction of the premolars, canine, and incisors. As a result, it was considered that the amount of distal tipping of the maxillary second molar was lower than the planned amount [12].

## 5. Limitations

First of all, there were some inherent limitations to the design of this study. Retrospective studies have some disadvantages. This type of study is vulnerable to selection bias and information bias as a result of its retrospective nature, which can compromise its validity. However, the criteria in this study were established very carefully and thoroughly. Consequently, the number of subjects that met all the criteria was minimal, and they were all included in this study. Another risk of bias in retrospective studies is the limited ability to control patient compliance. However, patient compliance in wearing the aligners and elastics, including attendance at the dental appointments, was verified from the patients’ chart records, reducing the risk of this issue.

Next, methods and procedures for calculating tooth movements and reference points in the ClinCheck program are not disclosed due to the company’s confidential information. However, the cephalometric measurements in this study used reference points, reference planes, and measuring methods specifically chosen to support accurate cephalometric analysis. Consequently, discrepancies could have occurred in the experimental results if the measuring methods and reference locations used in this study and the ClinCheck program differed from each other.

Lastly, the results may not be applicable across all age groups. According to several studies, age affects the rate of orthodontic tooth movement. The rate of tooth movement in adults is significantly slower than in adolescents [34]. Due to the delayed initial cellular response, responsiveness to orthodontic force in older patients might be lower than in younger groups [35]. This study selected an adult population aged 19 to 60 years as the subjects to reduce factors that affect the performance of clear aligners such as skeletal growth and rate of bone remodeling. Therefore, treatment outcomes may differ when applied to other age groups, such as adolescents and senior adults

## 6. Suggestions

For a better understanding of maxillary molar distalization when using clear aligners, further research is necessary. Firstly, the number of samples should be increased. Since confidence in the result is more likely to increase with a larger sample size, a higher sample size can increase the significance level of the findings and reflect the behavior of an entire group more accurately.

Secondly, assessments of dental movements with Invisalign should be conducted in combination with cone-beam computed tomography (CBCT) because analyses of tooth movements, including the distance, angle of tipping, and angle of rotation, might be able to provide clearer details if appropriate reference points can be located.

Thirdly, molar distalization with clear aligners can impact the inclination of the occlusal plane. The distal movement of molars often involves forces that can induce tipping and rotational movements, which, in turn, affect the occlusal plane [31]. It would be beneficial for future research to investigate how the inclination of the occlusal plane changes following distalization.

Lastly, future studies should assess patients who receive treatment with maxillary molar distalization combined with anchorage reinforcement by orthodontic mini-screws. Since mini-screws provide increased anchorage capacity, they may affect the treatment outcome.

## 7. Conclusions

- This preliminary study revealed that the actual distal translation of maxillary molars achieved using clear aligners with sequential molar distalization was significantly less than the planned distal translation.
- No significant differences were detected between the planned and actual angulation movements.
- The distal tipping of maxillary second molars was greater than planned distal tipping, while the distal tipping of maxillary first molars was less than planned distal tipping.
- To improve the predictability of maxillary molar movement, overcorrection or auxiliary anchorage devices may be required.

## Figures and Tables

**Table 1:** Demographic data of the sample.

Categories	Total *n* = 14		Mean ± SD	Min-Max
Sex	Male 4	Female 10		
Age (years)			33.61 ± 8.56	20–49
Numbers of aligners			49.14 ± 14.02	32–79

Min, minimum; Max, Maximum.

**Table 2:** Descriptive statistics.

Tooth Movement	N	Planned					Actual				
		Mean	SD	Median	Min	Max	Mean	SD	Median	Min	Max
U6_MDT (mm)	14	2.23	0.27	2.25	1.60	2.60	0.91	0.28	0.99	0.43	1.43
U7_MDT (mm)	14	2.43	0.53	2.35	1.90	4.10	0.86	0.25	0.81	0.52	1.40
U6_MDA (degree)	14	2.36	2.53	2.35	−2.10	6.50	0.96	3.50	0.25	−3.50	7.75
U7_MDA (degree)	14	−0.35	4.42	−0.25	−12.10	4.90	1.63	3.88	1.88	−6.75	7.00

U6, maxillary first molar; U7, maxillary second molar; MDT, mesiodistal translation; MDA, mesiodistal tipping. Positive values were assigned for distal translation/angulation. Negative values were assigned for mesial translation/angulation. Min, minimum; Max, Maximum.

**Table 3:** Difference in means between planned and actual maxillary molar movements.

Tooth Movement	Planned		Actual		Mean Difference (Planned–Actual)		
	Mean	SD	Mean	SD	Mean	SD	*p*-Value
U6_MDT (mm)	2.23	0.27	0.91	0.28	1.32	0.42	0.000 *
U7_MDT (mm)	2.43	0.53	0.86	0.25	1.57	0.45	0.000 *
U6_MDA (degree)	2.36	2.53	0.96	3.50	1.39	4.83	0.300
U7_MDA (degree)	−0.36	4.42	1.63	3.88	−1.98	5.66	0.212

* Statistical significance at *p* ≤ 0.05. A negative value indicated that the actual values had greater distal movements than the planned values.

## Data Availability

Data are contained within the article.

## References

[1] Proffit W.R., Fields H.W. (2013). Contemporary Orthodontics.

[2] Richmond S., Shaw W.C., O’Brien K.D., Buchanan I.B., Jones R., Stephens C.D., Roberts C.T., Andrews M. (1992). The development of the PAR Index (Peer Assessment Rating): Reliability and validity. Eur. J. Orthod..

[3] Borzabadi-Farahani A. (2012). A review of the evidence supporting the aesthetic orthodontic treatment need indices. Prog. Orthod..

[4] Brook P.H., Shaw W.C. (1989). The development of an index of orthodontic treatment priority. Eur. J. Orthod..

[5] Gianelly A.A. (1998). Distal movement of the maxillary molars. Am. J. Orthod. Dentofac. Orthop..

[6] Gianelly A.A. (1998). A strategy for nonextraction Class II treatment. Semin. Orthod..

[7] Kinzinger G.S., Eren M., Diedrich P.R. (2008). Treatment effects of intraoral appliances with conventional anchorage designs for non-compliance maxillary molar distalization: A literature review. Eur. J. Orthod..

[8] Su H., Han B., Li S., Na B., Ma W., Xu T.M. (2014). Factors predisposing to maxillary anchorage loss: A retrospective study of 1403 cases. PLoS ONE.

[9] Rossini G., Parrini S., Castroflorio T., Deregibus A., Debernardi C.L. (2015). Efficacy of clear aligners in controlling orthodontic tooth movement: A systematic review. Angle Orthod..

[10] Zheng M., Liu R., Ni Z., Yu Z. (2017). Efficiency, effectiveness and treatment stability of clear aligners: A systematic review and meta-analysis. Orthod. Craniofac. Res..

[11] Tai S. (2018). Clear Aligner Technique.

[12] Dai F.F., Xu T.M., Shu G. (2019). Comparison of achieved and predicted tooth movement of maxillary first molars and central incisors: First premolar extraction treatment with Invisalign. Angle Orthod..

[13] Inchingolo A.M., Inchingolo A.D., Carpentiere V., Del Vecchio G., Ferrante L., Di Noia A., Palermo A., Di Venere D., Dipalma G., Inchingolo F. (2023). Predictability of Dental Distalization with Clear Aligners: A Systematic Review. Bioengineering.

[14] Galan-Lopez L., Barcia-Gonzalez J., Plasencia E. (2019). A systematic review of the accuracy and efficiency of dental movements with Invisalign(R). Korean J. Orthod..

[15] Papadimitriou A., Mousoulea S., Gkantidis N., Kloukos D. (2018). Clinical effectiveness of Invisalign(R) orthodontic treatment: A systematic review. Prog. Orthod..

[16] Ravera S., Castroflorio T., Garino F., Daher S., Cugliari G., Deregibus A. (2016). Maxillary molar distalization with aligners in adult patients: A multicenter retrospective study. Prog. Orthod..

[17] Bae E.J., Kwon H.J., Kwon O.W. (2014). Changes in longitudinal craniofacial growth in subjects with normal occlusions using the Ricketts analysis. Korean J. Orthod..

[18] Roth R.H. (1987). The straight-wire appliance 17 years later. J. Clin. Orthod..

[19] McNamara J.A. (1984). A method of cephalometric evaluation. Am. J. Orthod..

[20] Bjork A. (1955). Facial growth in man, studied with the aid of metallic implants. Acta Odontol. Scand..

[21] Bjork A., Skieller V. (1977). Growth of the maxilla in three dimensions as revealed radiographically by the implant method. Br. J. Orthod..

[22] Mishra P., Pandey C.M., Singh U., Gupta A., Sahu C., Keshri A. (2019). Descriptive statistics and normality tests for statistical data. Ann. Card. Anaesth..

[23] McCluskey A., Lalkhen A.G. (2007). Statistics II: Central tendency and spread of data. Contin. Educ. Anaesth. Crit. Care Pain.

[24] Cronbach L.J. (1951). Coefficient alpha and the internal structure of tests. Psychometrika.

[25] Morton J., Derakhshan M., Kaza S., Li C. (2017). Design of the Invisalign system performance. Semin. Orthod..

[26] Chiu P.P., McNamara J.A., Franchi L. (2005). A comparison of two intraoral molar distalization appliances: Distal jet versus pendulum. Am. J. Orthod. Dentofac. Orthop..

[27] Ngantung V., Nanda R.S., Bowman S.J. (2001). Posttreatment evaluation of the distal jet appliance. Am. J. Orthod. Dentofac. Orthop..

[28] Yurdakul Z., Karsli N. (2024). Evaluating anchorage loss in upper incisors during distalization of maxillary posterior teeth using clear aligners in adult patients: A prospective randomized study. Korean J. Orthod..

[29] Fontana M., Cozzani M., Caprioglio A. (2012). Soft tissue, skeletal and dentoalveolar changes following conventional anchorage molar distalization therapy in class II non-growing subjects: A multicentric retrospective study. Prog. Orthod..

[30] Garino F., Castroflorio T., Daher S., Ravera S., Rossini G., Cugliari G., Deregibus A. (2016). Effectiveness of Composite Attachments in Controlling Upper-Molar Movement with Aligners. J. Clin. Orthod..

[31] Simon M., Keilig L., Schwarze J., Jung B.A., Bourauel C. (2014). Treatment outcome and efficacy of an aligner technique--regarding incisor torque, premolar derotation and molar distalization. BMC Oral Health.

[32] D’Anto V., Valletta R., Ferretti R., Bucci R., Kirlis R., Rongo R. (2023). Predictability of Maxillary Molar Distalization and Derotation with Clear Aligners: A Prospective Study. Int. J. Environ. Res. Public Health.

[33] Caruso S., Nota A., Ehsani S., Maddalone E., Ojima K., Tecco S. (2019). Impact of molar teeth distalization with clear aligners on occlusal vertical dimension: A retrospective study. BMC Oral Health.

[34] Alikhani M., Chou M.Y., Khoo E., Alansari S., Kwal R., Elfersi T., Almansour A., Sangsuwon C., Al Jearah M., Nervina J.M. (2018). Age-dependent biologic response to orthodontic forces. Am. J. Orthod. Dentofac. Orthop..

[35] Schubert A., Jager F., Maltha J.C., Bartzela T.N. (2020). Age effect on orthodontic tooth movement rate and the composition of gingival crevicular fluid: A literature review. J. Orofac. Orthop..

